# Detection of novel 3' untranslated region extensions with 3' expression microarrays

**DOI:** 10.1186/1471-2164-11-205

**Published:** 2010-03-26

**Authors:** Lieven Thorrez, Leon-Charles Tranchevent, Hui Ju Chang, Yves Moreau, Frans Schuit

**Affiliations:** 1 Gene Expression Unit, Department of Molecular Cell Biology, Katholieke Universiteit Leuven, Leuven, Belgium; 2 SymBioSys, K.U.Leuven Center for Computational Systems Biology, Leuven, Belgium; 3 SCD-ESAT, Department of Electrical Engineering, Katholieke Universiteit Leuven, Leuven, Belgium

## Abstract

**Background:**

The 3' untranslated regions (UTRs) of transcripts are not well characterized for many genes and often extend beyond the annotated regions. Since Affymetrix 3' expression arrays were designed based on expressed sequence tags, many probesets map to intergenic regions downstream of genes. We used expression information from these probesets to predict transcript extension beyond currently known boundaries.

**Results:**

Based on our dataset encompassing expression in 22 different murine tissues, we identified 845 genes with predicted 3'UTR extensions. These extensions have a similar conservation as known 3'UTRs, which is distinctly higher than intergenic regions. We verified 8 of the predictions by PCR and found all of the predicted regions to be expressed. The method can be extended to other 3' expression microarray platforms as we demonstrate with human data. Additional confirming evidence was obtained from public paired end read data.

**Conclusions:**

We show that many genes have 3'UTR regions extending beyond currently known gene regions and provide a method to identify such regions based on microarray expression data. Since 3' UTR contain microRNA binding sites and other stability determining regions, identification of the full length 3' UTR is important to elucidate posttranscriptional regulation.

## Background

The 3' untranslated region (3'UTR) of a gene does not belong to the protein coding sequence, however it plays an important role in posttranscriptional regulation [1]. This region of the transcript typically contains binding sites for proteins and microRNAs which influence the stability, localization and translation of the messenger RNA [2]. Not only the presence of microRNAs and RNA binding proteins themselves determines the mRNA fate, but also the presence of their binding sites on the transcript is critical for the specific regulation to occur. Therefore it is important to identify the 3' ends of the transcript.

Delineation of the 3' end of a transcript so far relied on ESTs or high-throughput sequencing of full-length cDNAs in various cell lines or in one specific tissue [3,4]. Paired-End diTag (PET) analysis, during which short fragments from both transcript ends are extracted, concatenated and sequenced, possesses a unique capability to accurately and efficiently characterize transcript boundaries. This approach was demonstrated on 2 human cancer cell lines [5]. However it has recently been shown that 3'UTR length varies across tissues types and is highly dependent upon cell division [6], so many cell lines, which inherently are dividing cells, may not express the full length 3'UTR. A direct consequence is that many of the current annotations do not reflect the potential full length 3' UTR. Indeed, by use of RACE (rapid amplification of cDNA ends), analyzing both 5' and 3' ends and then hybridizing the products to whole genome tiling arrays, it was recently demonstrated that, for most of the genes, there are RNAs that extend well beyond known gene borders, often further than three megabases away [7].

Affymetrix genechips are widely used microarray platforms to measure expression of thousands of transcripts simultaneously. With this technology, mRNAs extracted from the experimental sample are labeled and then hybridized to 25 bp long probes [8]. During the array design process [9], Affymetrix collected sequences and annotations from various public databases including GenBank, dbEST and RefSeq and clustered these sequences. The longest sequence in each cluster was used as the representative for that cluster, with preference given to RefSeq sequences. Pair-wise alignment of the probe sequences against the non-redundant mRNA cluster was used to assign probe sets to transcripts. Each probe, through its specific 25 bp sequence, is designed to target one transcript although for some probes, cross-hybridization to other transcripts is known. In order to get a better estimation of the expression level of a transcript, probes are grouped per 11 probe pairs, known as probe sets [10]. The signal of a probe set is not only a factor of the transcript abundance, but also depends upon the position of the probe set. At equal GC content, the more 3' of a transcript the probe set is designed, the higher the signal will be, since the labeling protocol initiates with an oligo d(T) primer. Therefore, probes have been designed to recognize the 3' ends of transcripts, which comprises mostly the 3' untranslated region. Affymetrix 3' expression arrays were designed years ago based on EST data, which at that time were largely unannotated (e.g., June 2002 for the mouse 430 2.0 array). Currently, many probe sets are still not assigned to any RefSeq transcript while their expression signal is clearly above the noise.

We developed a method which detects currently unannotated probe sets that target putative extensions of the known 3' UTRs. This algorithm makes use of expression data in many diverse tissues and can be applied to any 3' expression platform where such data are available.

## Results

### Mouse gene expression data

We have focused our analysis on the Affymetrix 430 2.0 mouse expression array (MOE430 2.0), since this is a widely used platform, containing 45101 probe sets covering most of the known RefSeq transcripts. Our dataset consists of 70 microarrays in total covering 22 different murine tissues with 3-5 replicates per tissue. We covered a wide range of tissue types that included slow dividing tissues such as brain cortex and eye as well as fast dividing tissues such as small intestine and embryonic stem cells.

Of the 45101 probe sets, 5400 (12%) currently do not correspond to any known RefSeq gene (august 2008), but are expressed significantly above the noise level in at least one of our arrays.

### Computational screen for extended probe sets

**Figure 1:**
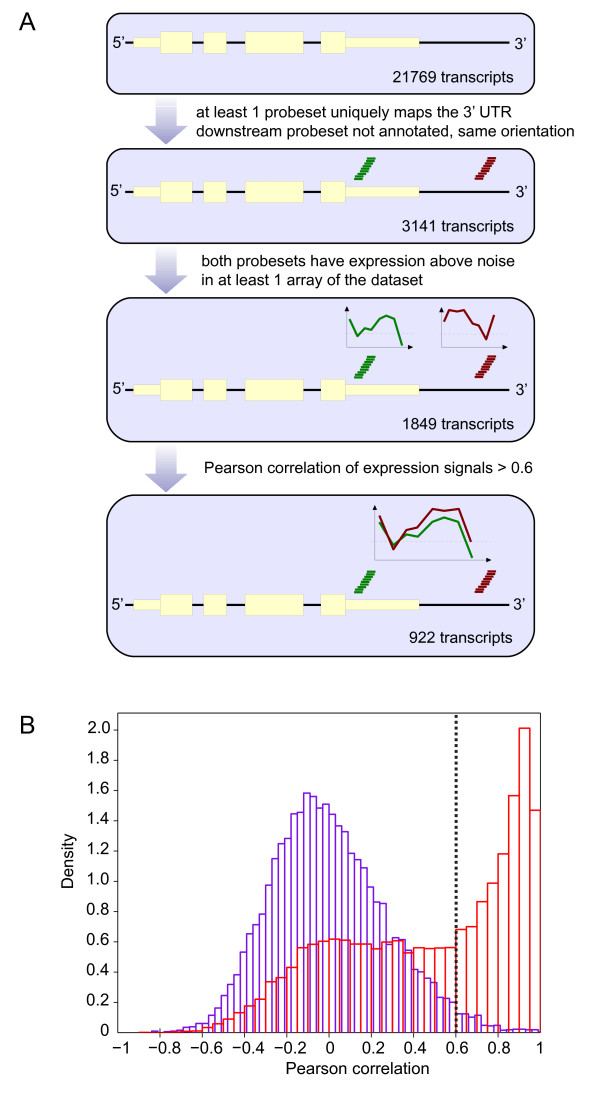
**Identification of unannotated extended probe sets**. **A**. At the core of this analysis is our murine mRNA expression database comprising 22 different tissues. Unannotated probe sets binding putative 3' UTR extensions were identified as shown in the flow chart. The number of RefSeq transcripts retained after each filtering step is shown, finally resulting in 922 transcripts corresponding to 845 unique gene symbols for which 3' UTR extensions are predicted. **B**. Histogram with Pearson's correlations. The red histogram depicts correlations between the 1849 probe set pairs selected as shown in panel A. The blue histogram depicts correlations between random pairs of probe sets from the same microarray platform. As a cut-off for statistically significant co-expression, a Pearson correlation of 0.6 was chosen, resulting in an estimated false positive rate of 2%.

As a starting point of our algorithm, RefSeq was chosen because of the high quality curation and up-to-date annotations. For each of the 21769 murine transcripts in RefSeq, the algorithm detects the probe set located most 3'of the transcript (termed hereafter 'primary probe set') and then evaluates the next downstream probe set whether or not it might target an extended transcript. The analysis pipeline is depicted in Figure 1A. Only probe set pairs are considered where the downstream probe set binds the same strand and is not yet annotated to a RefSeq transcript. We removed 6808 probe sets from the analysis because these map to multiple locations on the mouse genome; this resulted in 38293 probe sets for further analysis. We identified 3141 transcripts which had a probe set pair. To eliminate correlations based on noisy expression, we only considered probe sets which have a log2 expression signal >6 in at least one array of the entire dataset. From the 3141 RefSeq transcripts, 1849 (59%) were found to be covered by co-oriented probe sets which generated expression signals above noise.

To determine consistent co-expression, we calculated the Pearson correlation coefficients between the expression profiles for all probe set pairs retained thus far and plotted the histogram (Figure 1B, red bars). To select a statistically relevant threshold for Pearson correlation, we generated Pearson correlation coefficients of pairs randomly picked in the dataset that also have a signal above the noise (blue bars). By choosing a correlation cut-off value of 0.6, the right tail of the blue histogram contains 2.18% of the random observations, which is the estimated false positive rate. As an additional way to estimate the false positive rate for each individual primary probe set, we calculated the correlation of this probe set with all uniquely mapping probe sets on all chromosomes except the chromosome to which the probe set mapped. The stringent assumption being that a correlation of two probe sets on different chromosomes represents a false positive (meaning that the transcript does not extend over different chromosomes). For each probe set, correlations were calculated on average over 27340 other probe sets. The percentage of probe sets with a correlation >0.6 was 1.84% on average (median 1.32%), with a total interval ranging from 0.022-8.60%, which is consistent with the previous false positive estimate.

Of the 1849 probe set pairs identified in the first step of the analysis, 922 pairs (50%) had a Pearson coefficient higher than 0.6. We mapped these 922 transcripts to 845 unique gene symbols. For genes with several transcript isoforms, we selected the transcript of which the current annotation extended most 3'. These 845 genes are listed in Additional file 1, Table S1. Taking into account previous estimates, we expect between 15 and 18 out of 845 extended probe sets to be false positives.

**Figure 2:**
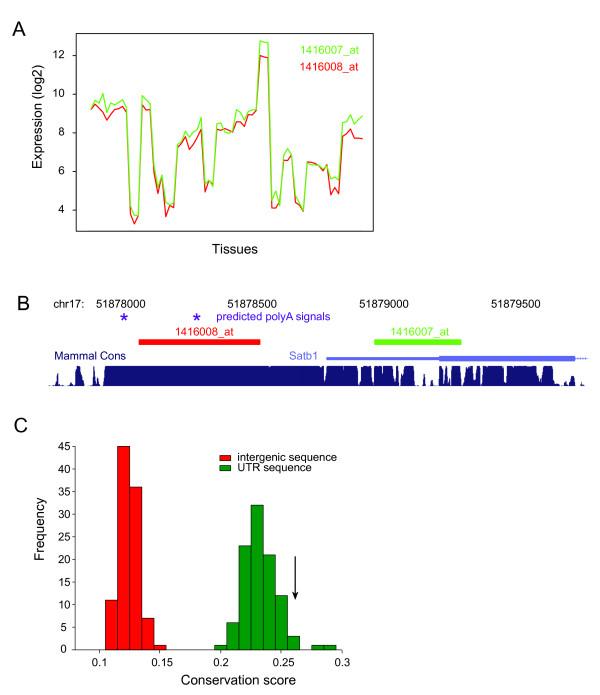
**Expression and sequence conservation of extended probesets**. **A**. An example of two probe sets with concordant expression profiles. Expression of the 2 correlating probe sets is shown across all 70 microarrays, which are 3-5 biological replicates from 22 different tissues. **B**. Genomic context of the probe sets shown in panel A. Note that transcriptional direction is from right to left (negative strand). The red probe set (1416008_at) is targeting an intergenic region, which according to our algorithm likely is an extended 3'UTR of the upstream gene *Sat1b*. The region immediately downstream of the known 3' UTR; this region is highly conserved and contains 2 predicted polyA signals (indicated by stars). **C**. Conservation score distribution of mouse 3' UTR regions (green) and intergenic regions (red). Black arrow indicates the conservation score of the extended regions.

As an example of probe set correlation, the log2 expression signals of probe sets 1416007_at and 1416008_at are shown in Figure 2A. 1416007_at is annotated to target the 3' end of a *Satb1 *(special AT-rich sequence binding protein 1), whereas 1416008_at is 0.7 kb downstream of this gene and according to information displayed in the UCSC genome browser targets an intergenic region (Figure 2B). It can be observed that the expression signal of both probe sets is nearly identical, with only a consistent shift in expression signal intensity over the different microarrays of various tissues. The genomic space between the two probe sets is very well conserved over mammals, which likely indicates the presence of functional regulatory regions. Such conservation was observed for many of the predicted 3'UTR extended regions, as we demonstrate below. Of interest, the predicted extension for *Satb1 *is in perfect concordance with the occurrence of two polyA sites after the current annotated end of the transcript [11]. The region between the annotated end and the extended probe set (709 bp) contains the first polyA site and the second polyA site is positioned immediately downstream of the probe set. Since detection of the transcript usually is better the closer a probe sets is relative to the 3' end, many of the extended probe sets provide better markers for gene expression. An example of this is provided in Additional file 2, Figure S1A, where the extended probe set for gene *D10Bwg1379e *shows a clear expression signal in a number of samples which would be in the background range when using the annotated probe set.

### In silico validation of predicted extensions

For a significant fraction of the extended 3'UTR probe sets, we could find confirmation by gene prediction methods. In Ensembl, a wide range of methods including ab initio gene predictions, homology and gene prediction hidden Markov models were used to make gene predictions. These 'Ensembl genes' are regarded as being accurate predicted gene structures with a low false positive rate, since they are all supported by experimental evidence of at least one form via sequence homology [12]. 197 of the probe sets detecting extended 3'UTRs were residing in these regions predicted by Ensembl. These genes are indicated in Additional file 1, Table S1 with an asterisk in the last column. An example is shown for *Lpin2*, corresponding to the Ensembl predicted gene ENSMUST00000112649 (Additional file 2, Figure S1B). Also, although not annotated as such by RefSeq nor UCSC genes, 598 probe sets when queried within NetAffx [13] (the online Affymetrix analysis center) were assigned to the genes we predicted. When this information could be found, this was indicated next to the probe set in Additional file 1, Table S1.

A consequence of the existence of extended 3'UTR transcripts is that probe sets which were thought of binding at the 3' end may bind more upstream of the transcript. Labeling efficiency of Affymetrix 3' expression arrays decreases as a function of distance from the polyA tail and thus the signal of such a probe set may be relatively low and not meet our expression signal threshold. Therefore we also looked at consecutive probe set pairs where the secondary probe set met the expression criterion while the primary did not and where probe sets had a Pearson correlation exceeding 0.6. This resulted in an additional 44 probe sets putatively targeting extended transcripts. As an example, expression profiles of probe set 1439965_at, downstream of probe set 1456940_at targeting *Slc43a2 *are depicted in Additional file 2, Figure S1C. It can be observed that the downstream probe set (although by current knowledge thought to be targeting intergenic sequence) has higher expression values than the gene-targeting probe set. However, since the false positive rate will be higher in this set of probe sets due to more noisy expression data, we chose not to include these in our list of putatively extended probe sets.

The distance between the extended probe sets and the primary probe set ranged from 76 base pairs to 500 kb. The distribution of the distances is shown in Additional file 3, Figure S2. The majority of the extensions (600/845) were less then 2 kb. However, a small fraction (39/845 = 5%) of the probe set pairs was more than 10 kb apart. Most likely, correlated probe sets located far away on the genome arise from a 3'UTR which has been spliced. Splicing over longer genomic distances has been described [7]. A remote possibility exists that a correlation between two consecutive probe sets was caused by a hitherto unknown gene located downstream of the putatively extended gene that would have a similar expression profile. To exclude as much as possible regions that get spliced out, motif finding was limited to the predictions for extended regions <1 kb (n = 338). In order to classify the extended regions as either 3' UTR or intergenic sequence, we looked for motif enrichment of 3'UTRs versus intergenic regions. We scanned for two types of known stability elements: C-rich and AU-rich stability elements and for 2 types of polyA elements: the classical mammalian and the cytoplasmic polyA element. In addition, we investigated the number of microRNA binding sites predicted by the Targetscan algorithm [14]. For the microRNA target prediction, conservation scores were not taken into account. For both known 3'UTR regions and intergenic regions, 100 random datasets were generated containing the same amount of sequence as the extended regions. Results are shown in Additional file 4, Table S2. Based on occurrence of these motifs, we were not able to discriminate known 3'UTRs from intergenic sequence. Therefore these data could not be used to confirm the predictions for the extended regions.

We could however discriminate 3'UTR regions from intergenic regions based on phylogenetic conservation scores. Conservation scores were calculated with the PhastCons method based on the multiple alignments of 20 placental mammalian genomes. PhastCons utilizes a hidden Markov model which estimates the probability that each nucleotide belongs to a conserved element and is effective for picking out conserved elements [15]. For each of the 100 sets (known 3'UTR and intergenic sequence) an average conservation score was calculated. Results are depicted in Figure 2C. Intergenic regions are poorly conserved, with an average conservation score of 0.124, while 3' UTRs display a more elevated conservation with an average of 0.233. Average conservation of the predicted extensions was 0.26, which clearly is outside the range of our 100 set of intergenic sequences (min 0.108 - max 0.152), but well within that of the known 3' UTRs (min 0.202 - max 0.286). With a normal distribution Z-statistic, we therefore propose that the predicted 3' UTR extensions are similar to the known 3' UTRs with p < 0.0001 (taken from the intergenic distribution).

### Wet lab validation of predicted extensions

**Figure 3:**
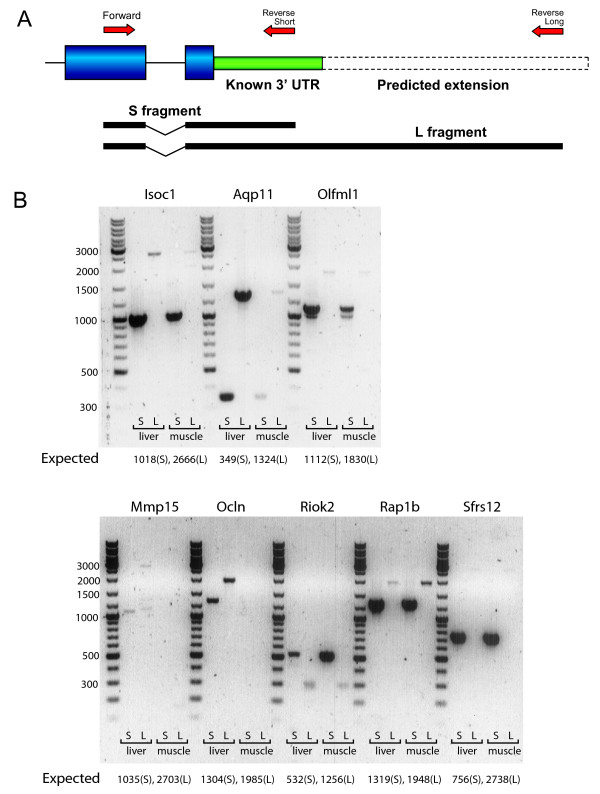
**Detection of transcripts containing the predicted 3'UTR extension**. **A**. Schematic overview of the validation PCR setup. For each of the eight tested genes, two primer pairs were designed with the forward primer in common. The reverse primer was binding either within the known 3' UTR region (reverse short) or in the predicted extension (reverse long). Amplified regions were termed S (short) or L (long), respectively and the expected size of these fragments for each of the genes is displayed below. In case of false positive prediction, no PCR fragment is expected for the L fragment, since the reverse long primer then has no template to bind to. Contamination of genomic DNA was excluded because primer pairs were spanning at least one intron. **B**. Results of PCR amplification visualized by gel electrophoresis. For each gene, four lanes represent amplification of the short and long fragment in two tissues: liver and muscle. Next to these four lanes a size marker was included with corresponding fragment sizes indicated left of the image.

To confirm the existence of the predicted 3'UTR extensions, we performed PCR assays with specific primer pairs amplifying the known 3' UTR and the extended 3'UTR. Primer pairs were designed such that the forward primer was common for the annotated and extended 3'UTR and we assured that at least one intron was present inside the amplified fragment, to exclude amplification of genomic DNA. A general design of this experiment is depicted in Figure 3A. We randomly chose eight genes with a predicted 3'UTR extension less than 1 kb for this validation and performed PCR on both liver and muscle cDNA. Results are shown in Figure 3. For all eight genes, we could verify expression of the extended 3'UTR as all of the long PCR fragments displayed a PCR fragment. The fragments corresponded to the expected lengths, except for one gene (*Riok2*) where the long fragment was much shorter than expected. For two genes *(Mmp15 *and *Ocln*), expression of both fragments was only detected in liver, which was consistent with a low expression value on the microarray (data not shown). Also, for some genes (*Isoc1, Olfml1, Mmp15*) next to the main fragment, a smaller and less abundant fragment was detected, which is likely to reflect minor splice variants. To verify that PCR products originated from the intended regions, we sequenced the PCR products from *Olfml1 *and *Riok2*. Sequencing revealed multiple alternative splicing products for both the known and extended 3'UTR of *Olfml1 *as shown in Additional file 5, Figure S3. Also, the shorter size of the *Riok2 *extended 3'UTR was confirmed to be due to an alternative splicing event, resulting in a 282 bp long fragment instead of the predicted 1256 bp.

### Cross-species application

The currently proposed method to predict 3' UTR extensions may be easily adapted to other microarray platforms. As a proof of principle, we applied the method we used for mouse 430 2.0 arrays to a dataset consisting of normal human tissue microarrays. A publicly available set of 64 Affymetrix U133 Plus2.0 arrays was used in a manner analogous to the murine tissue set. RefSeq contains 31270 human transcripts, of which 4662 are targeted by at least one probe set on the array and have an unannotated downstream probe set in the same orientation. After applying the expression threshold, 2126 probe sets pairs were retained. The Pearson correlation graph was similar to the murine data (Figure 1B, Additional file 6, Figure S4A), so a correlation threshold of 0.6 was applied resulting in a final prediction of 627 extended 3' UTR regions. The 627 human genes and probe set pairs are listed in Additional file 7, Table S4. On the set of 162 regions smaller than 1 kb, conservation scores were calculated with PhastCons based on the multiple alignment of 17 vertebrate species. Results are depicted in Additional file 6, Figure S4B. Similar to the murine data, conservation of the predicted extensions (average 0.2481) is clearly higher than intergenic sequence (average 0.082 ranging from 0.059 to 0.115) and even a little higher than known 3' UTR regions (average 0.188 ranging from 0.140 to 0.231). As a validation, we looked at transcript delineation by experimental evidence. The Gene Identification Signature Paired End diTag (GIS-PET) method provides sequence information from tags at both the 5' and 3' end of a transcript. Data from GIS-PET experiments on human embryonic stem cells and a few cell lines can be visualized and downloaded from the UCSC genome browser. An example of this is shown for *Afg3l2 *in Additional file 6, Figure S4C. Tags can be seen where the RefSeq transcript ends, but also downstream of this. The extended probe set targets the region in between, which is a highly conserved region. Of the 162 sequences < 1 kb, 65 (40%) were confirmed by tags mapping on the target region of the extended microarray probe set or even further downstream.

## Discussion

It was suggested previously that Affymetrix GeneChips have the potential to be used as a discovery tool for exploring the exotic transcriptome, and go beyond their standard use as a measure of mRNA for protein-coding genes [16]. In this work, we have developed a method to use 3'UTR targeted expression arrays to investigate the current annotations of 3' ends. We looked at unannotated probe sets downstream of annotated genes and compared the expression profiles of both probe sets over a large set of arrays from different murine tissues. With this approach, we found that 845 murine genes display expression downstream of the RefSeq annotation. Some of these genes have been studied extensively before but sparse evidence of an extended 3'UTR could be found in the literature. One example is the gene that encodes the GLP1-receptor (*Glp1r*), for which Northern blot experiments resulted in two distinct mRNA lengths [17].

The finding of 3'UTRs extending further than currently annotated has important implications for data analysis. First, Affymetrix 3' expression probe sets have been designed within 600 bp of 3' ends, to ensure an efficient labeling. An important consequence of the unknown extended 3'UTRs is that the probe set which was previously thought to be close to the 3' end might not give a high signal since the real 3' end is further away and thus a lower labeling efficiency occurred. This was possibly the case for the additional 44 probe sets which we found correlated but where the signal generated with the proximal probe set was too low for inclusion in the dataset. Therefore, transcripts where only the annotated (proximal) probe set is considered, might give false negative results, attributed to low expression, but in fact caused by the extended 3'UTR. Extended probesets detecting longer 3'UTRs may provide a higher expression signal, but have an increased chance to give a background signal in the case the transcript is alternatively terminated. However, as for the current set of predictions, alternative termination is unlikely in the majority of the samples, since the extended probesets are selected based on a good coexpression profile with the primary probesets. If alternative termination would occur commonly, the extended probeset would not be retained in this analysis. The extended probe sets we describe in this paper are thus more reliable markers for measuring expression of such genes in conditions where the full length 3' UTR is used.

Second, exon arrays are increasingly used to capture variation by alternative termination and alternative splicing. However, probes for these exon arrays have been mainly designed in the regions of the currently known exons. As a result, information on extended 3'UTRs, such as the ones described in this paper may not be obtained.

Third, microRNA target sites have been shown to be enriched towards the end of the 3'UTR [18,19]. Therefore, incomplete 3' UTR annotations may obscure an important fraction of microRNA binding sites.

Knowledge of the full 3'UTR sequence is not only important from a data analysis perspective but is also clinically relevant. It has been estimated that 3' UTRs are associated with about 0.2% of known disease-associated mutations [20] and it was noted that "this number is likely a conservative estimate, especially since it is emerging that the boundaries of this regulatory region are still unknown for many genes in various tissues". Mutations in the 3' UTR region have been associated with many diseases such as systemic lupus erythematosus [21,22], spastic paraplegia [23] and cardiomyopathy [24]. A naturally occurring trinucleotide insertion in the 3'UTR of mouse tumor necrosis factor alpha (*Tnf*) mRNA has been shown to hinder RNA-binding proteins, thereby reducing mRNA stability [25]. Mice carrying this polymorphism have macrophages that are low producers of TNFalpha protein when stimulated with interferon gamma, highlighting the physiological importance of 3'UTR elements and their control of mRNA stability. Not only mutations, but also alternative splicing in the 3'UTR can lead to disease as was described for the Wiskott-Aldrich syndrome[26]. Alternative splicing is not necessarily linked to disease though, e.g. differential splicing within the 3'UTR of *Col17a1 *(type XVII collagen) produces two mRNA transcripts that differ 610 nucleotides in length in normal keratinocytes [27]. Expression of different transcripts coding for the same protein but varying in 3'UTR composition can also be used by the cell to control protein levels. Indeed this has been described for β-catenin (*Ctnnb1*), which has 3 mRNA splice variants that differ solely in their 3'UTRs due to alternative splicing or retaining of an intron [28]. Length of 3'UTR also provides translational control during developmental processes such as stem-cell proliferation, sex determination, neurogenesis and erythropoiesis [29]. Another consequence of the extended transcripts is that genes in a tail-tail orientation may overlap with their 3'UTR in an even greater number of cases than currently known. The function of many overlaps is antisense regulation and this occurs preferentially in 3' UTRs [30].

The number of genes described in this paper which have a longer 3'UTR than currently known is likely an underestimation for several reasons. First, it is dependent on probe sets designed by Affymetrix, which was based on limited EST information. Out of 21769 transcripts, 16592 have one probe set targeting the 3' UTR and only 3141 (14%) have another probe set targeting its putative extension. Second, we rely on a good concordance of probe set expression. Since our method selects only those probe sets which have a high correlation over 70 samples, we most likely did not detect many extended 3'UTRs which occur in just a few tissues.

We investigated the regulatory potential of the extended regions. To minimize the inclusion of intronic sequence, only extensions <1 kb away from the primary annotated probe set were considered for motif counting. However, based on counting of predicted stability elements, polyA signals or microRNA hits, we were not able to discriminate known 3'UTRs from intergenic sequence. Therefore occurrence of these motifs was not informative to classify the predicted extensions as 3'UTR or intergenic sequence. Next, we looked into evolutionary conservation of 3'UTRs versus intergenic sequences. Higher conservation scores can be observed in 3'UTR regions as compared to intergenic regions. This is logical because genomic changes in intergenic regions likely have less impact on selection than changes in genes. We found evidence of conservation of the predicted extended regions similar to that of known 3'UTRs, indicating that these regions have functional importance.

Most importantly, wet lab validation of 8 predicted extensions confirmed all of the *in silico *predictions. This is consistent with our prediction of the false positive rate (2%). For all of the extensions except one, we found the exact predicted lengths of PCR fragments in at least one tissue. As an exception, *Riok2 *had a region spliced out of the predicted extension, such that the final length of the extended 3'UTR was shorter than the known 3'UTR, which was confirmed by sequencing. Detailed sequencing analysis also revealed multiple alternative splicing products for the *Olfml1 *3'UTR.

Provided that large enough datasets of different tissues are available, this method can easily be applied to other microarray platforms. We demonstrated this by applying the method that we worked out for our own mouse microarray compendium to a publicly available human dataset. Using this approach, we identified 627 genes with putative extended 3'UTRs. Similar to mouse extended regions, these were also evolutionary conserved regions, clearly different from intergenic regions. Moreover, 40% of the regions <1 kb were confirmed by GIS-PET tags, which are short sequence tags derived from the beginning and end of a transcript. This figure is surprisingly high, since we expect only a subset of genes to be expressed in the cells used for these experiments (MCF7 and embryonic stem cells) and moreover, it is known that proliferating cells tend to express mRNAs with shortened 3' UTRs [6]. By sensitive detection of polyadenylation sites of genes in the ENCODE region encompassing 1% of the human genome, a large number of transcripts with 3' ends in introns or extragenic regions was detected, consistent with our observations [31].

## Conclusions

We established a method to investigate the 3'UTR extensions based on abundantly available microarray data. In our own mouse mRNA expression data set we observed that for 845 genes the 3' UTR was longer than annotated in Refseq. As the length of 3' UTR determines the regulatory elements which are present in the transcript, we believe that our observations have implications for the timing and amount of protein eventually synthesized. Knowledge of the full length of the 3'UTRs is therefore indispensable to fully understand posttranscriptional control.

## Methods

The expression dataset consisting of 70 microarrays covering 22 different murine tissues with 3-5 replicates per tissue was used as starting data. Data are accessible through the GEO database, with accession number GSE9954 [32]. The CEL files were analyzed using the affy library [33] of the BioConductor project [34] applying the Robust Multichip Average (RMA) function with default parameters (RMA background correction, probe-level quantile normalization and average difference summarization). During the normalization process, data were log2 transformed, and all further data analysis was performed on these log2 transformed data. As a threshold for expression above background, we used log2 (expression) >6 (expression signal > 64) in at least 1 array. This is a conservative estimate since about half of all expression values on every array are lower (data not shown).

Affymetrix annotation files for the MOE430 2.0 array were downloaded from NetAffx. Mouse genomic sequences and chromosomal coordinates were obtained from the UCSC Genome Browser [35] through the Galaxy software [36] using the UCSC July 2007 mm9 assembly, based on the NCBI Build 37. The affy package [33] from Bioconductor (version 2.1) [34] running under R (version 2.6.2) was used for the microarray data processing. Our algorithm is written in Perl (version 5.8.5) and is available upon request.

Sequence analysis for motif enrichment was performed with Perl. Motifs scanned for were: C-Rich Stability Element: YCCA 0...5 CCCW Y{0,4} TCYCC, AU-rich Stability Element: UAUUUAUWW, Cytoplasmic PolyA Element: UUUUAU 1.100 AAUAAA and Mammalian PolyA Element: A(A|U)UAAA 12.40 KKKKKKKKKK [5,0,0]. These motif descriptions were obtained from Transterm [37]. Scanning for predicted microRNA binding sites was performed with the Targetscan algorithm [14]. The sets containing known 3'UTRs and known intergenic sequences were obtained via Galaxy [38]. Conservation scores were extracted in Galaxy, selecting the phastCons30wayPlacental table (mouse) or phastCons17way (human) for the desired regions. Per set, averages of conservation scores were calculated. Screenshots were taken from the UCSC genome browser [35] and edited in Adobe Illustrator.

Sequences of PCR primers are shown in Additional file 8, Table S4. PCR was performed in a 25 μl volume with 0.2 μl Platinum Taq (Invitrogen) in Platinum Taq buffer, 300 nM primers (Sigma-Aldrich), 5 ng cDNA, 200 μM nucleotide mix (Fermentas) and 1.5 mM MgCl_2 _(Invitrogen). cDNA was prepared from the liver and gastrocnemius muscle of male C57Bl6 mice. Thermal cycling was performed on a GeneAmp PCR System 9700 (Applied Biosystems), 2' 94°C - 38 × (20" 94°C - 30" 60°C - 3' 72°C) - 10' 72°C - 4°C. Fragments were separated on a 1.5% agarose gel and visualized with SYBRSafe dye (Invitrogen), together with a Generuler DNA ladder (Fermentas). PCR products for *Olfml1 *and *Riok2 *were cut out from the gel, purified and sent out for sequencing to Makrogen. Sequences were aligned to the mouse genome by BLAT.

Normal human tissue data were retrieved from the Human Body Index (GSE7307, Neurocrine Bioscience, San Diego, California), of which a subset of 64 arrays were used, covering 18 different tissues with 3-5 replicates analogous to the murine dataset. Data were processed as described for the murine dataset.

## Authors' contributions

LT and LT participated in the design of the study, performed the computational analysis and validation experiments and wrote the paper. HJC performed computational analysis. YM acquired funding, assisted with the design of the study and provided general support. FS conceived of the study, participated in the design, supervised experiments, acquired funding and provided general support. All authors read and approved the final manuscript.

## Supplementary Material

Additional file 1 **Table S1**: List of Affymetrix mouse MOE 430 2.0 probe sets detecting extended 3'UTRs, obtained by stringent automated identification. Some probe sets were already predicted by Ensembl to target the extended 3'UTR, based on the presence of a longer human 3'UTR with high homology in the extended part. These probe sets are marked with an × in the appropriate column. Probe sets which are correctly annotated in Affymetrix' NetAffx are also indicated. Click here for file

Additional file 2 **Figure S1: Examples of correlated probesets**. **A**. Extended probe set 6 kb downstream of *D10Bwg1379e *gives better signals than the currently annotated probe set. Transcriptional direction is from right to left (negative strand). **B**. Confirmation of our extended probe sets by Ensembl gene predictions. *Lpin2 *is displayed with the RefSeq annotation (blue) and the Ensembl gene prediction (red). Transcriptional direction is from left to right (positive strand). **C**. Expression profiles of a probe set pair where the primary probe set has a low expression (log_2_expression <6) in all arrays. We found 44 similar cases on a genome-wide basis, but these were not retained for further downstream analysis in this study. Transcriptional direction of Slc43a2 is from left to right (positive strand). Click here for file

Additional file 3 **Figure S2: Histogram of the distances between the primary and extended probe sets**. The majority of predicted 3' UTR extensions are less than 2 kb. The graph is truncated at 10 kb but 5% of extensions are in the range of 10-500 kb. Click here for file

Additional file 4 **Table S2**: Occurrence of 3'UTR regulatory elements in the murine extended regions. Regions currently not annotated by Refseq to be part of the 3'UTR and < 1 kb were scanned for the occurrence of several known motifs. To classify predicted extensions as either 3' UTR or intergenic sequence, we repeated this analysis on 100 datasets of the same size randomly extracted from either known 3'UTR sequences or intergenic sequence. Each set contained 214657 bp. 95% confidence intervals are shown. Click here for file

Additional file 5 **Figure S3: Sequencing analysis of *Olfml1 *PCR products**. Both S (short, known 3'UTR) and L (long, extended 3'UTR) products revealed 3 different alternative splicing forms, indicated as S1-S3 and L1-L3. S1 and L1 are most abundant as can be seen on Figure 3B. Note that all sequencing products start more upstream in the last but one exon of *Olfml1*; this region is not depicted and only the alignment with the 3'UTR is shown. Click here for file

Additional file 6 **Figure S4: Detection of extended 3' UTRs on the Affymetrix human U133 Plus 2.0 platform**. **A**. Histogram with Pearson's correlations for human expression data. The red histogram depicts correlations between the 2126 probe set pairs before the final filtering step. The blue histogram depicts correlations between random probe sets. Similar to the mouse data, a Pearson correlation of 0.6 was chosen as a cut-off value. **B**. Human conservation score graphs, calculated with PhastCons based on the multiple alignment of 17 vertebrate species. Distributions in red and green represent intergenic and 3' UTR conservation respectively. Black arrow indicates the conservation score of the extended regions. **C**. GIS-PET track in the UCSC genome browser. GIS-PET tags are displayed in blue. Target regions for the Affymetrix U133 Plus 2.0 are indicated in black, PhastCons conservation score indicated in green. Click here for file

Additional file 7 **Table S3**: Sequences of PCR primers used for validation experiment Click here for file

Additional file 8 **Table S4**: List of Affymetrix human U133 Plus 2.0 probe sets detecting extended 3'UTRs, obtained by the same algorithm as applied to mouse data. Some probe sets were already predicted by Ensembl to target the extended 3'UTR and are marked with an × in the appropriate column. Probe sets which are correctly annotated in Affymetrix' NetAffx are also indicated. Click here for file
