# Supplementary figures and images for: Detection of novel 3' untranslated region extensions with 3' expression microarrays
Source: BMC Genomics. 2010 Mar 26;11:205. doi: 10.1186/1471-2164-11-205 (PMC2858751; doi:10.1186/1471-2164-11-205)

A

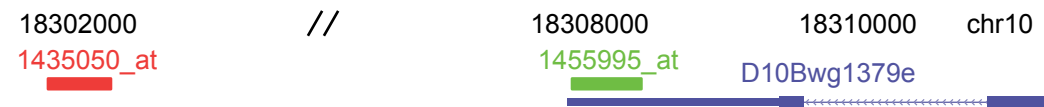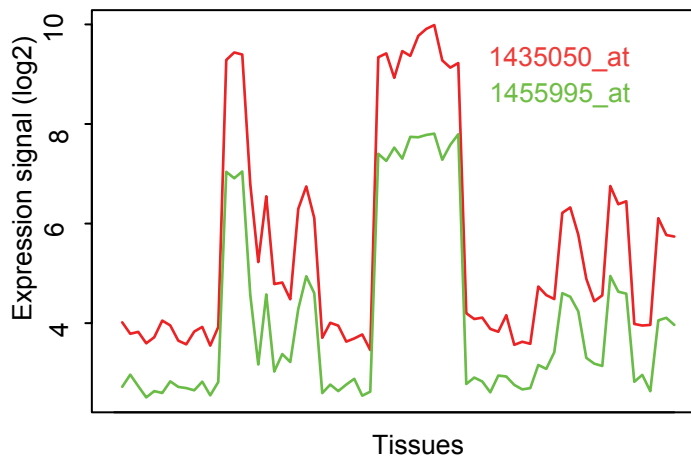

B

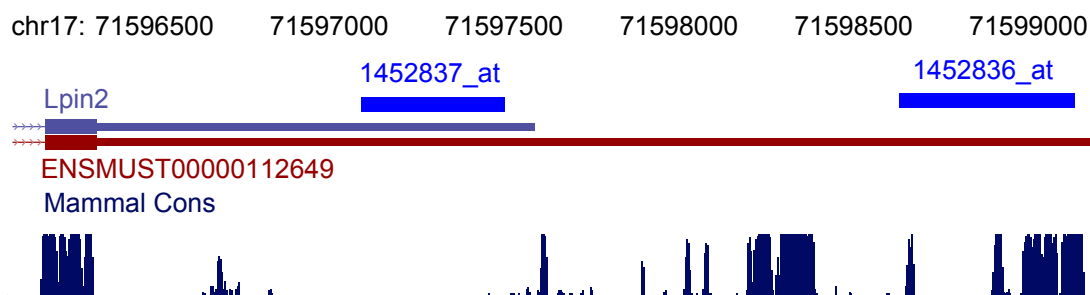

C

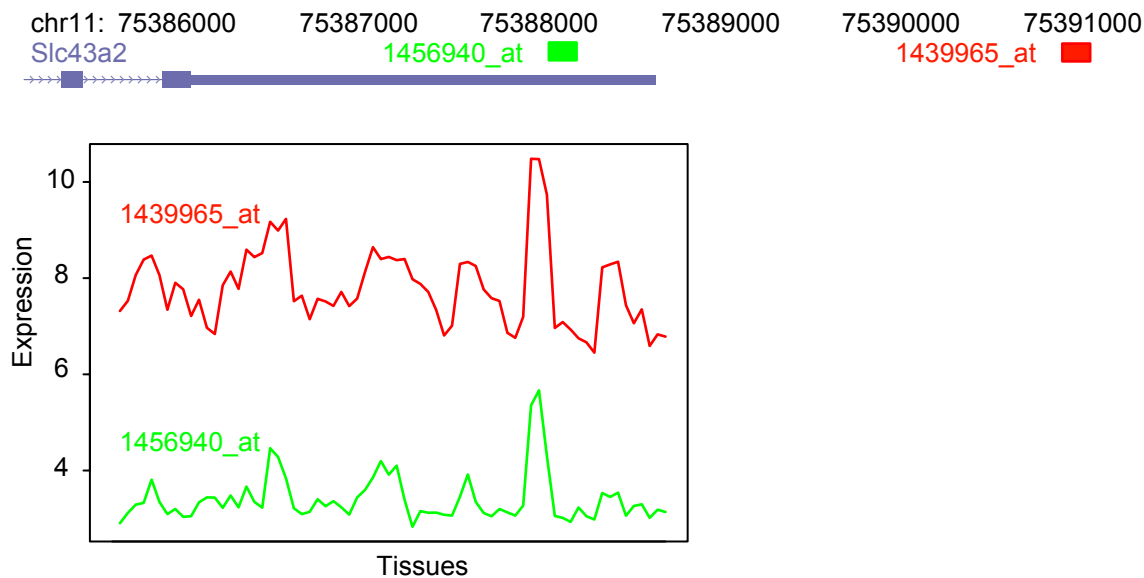

Supplement: Additional file 2 — Figure S1: Examples of correlated probesets. A. Extended probe set 6 kb downstream of D10Bwg1379e gives better signals than the currently annotated probe set. Transcriptional direction is from right to left (negative strand). B. Confirmation of our extended probe sets by Ensembl gene predictions. Lpin2 is displayed with the RefSeq annotation (blue) and the Ensembl gene prediction (red). Transcriptional direction is from left to right (positive strand). C. Expression profiles of a probe set pair where the primary probe set has a low expression (log2expression <6) in all arrays. We found 44 similar cases on a genome-wide basis, but these were not retained for further downstream analysis in this study. Transcriptional direction of Slc43a2 is from left to right (positive strand). [file 1471-2164-11-205-S2.PDF]

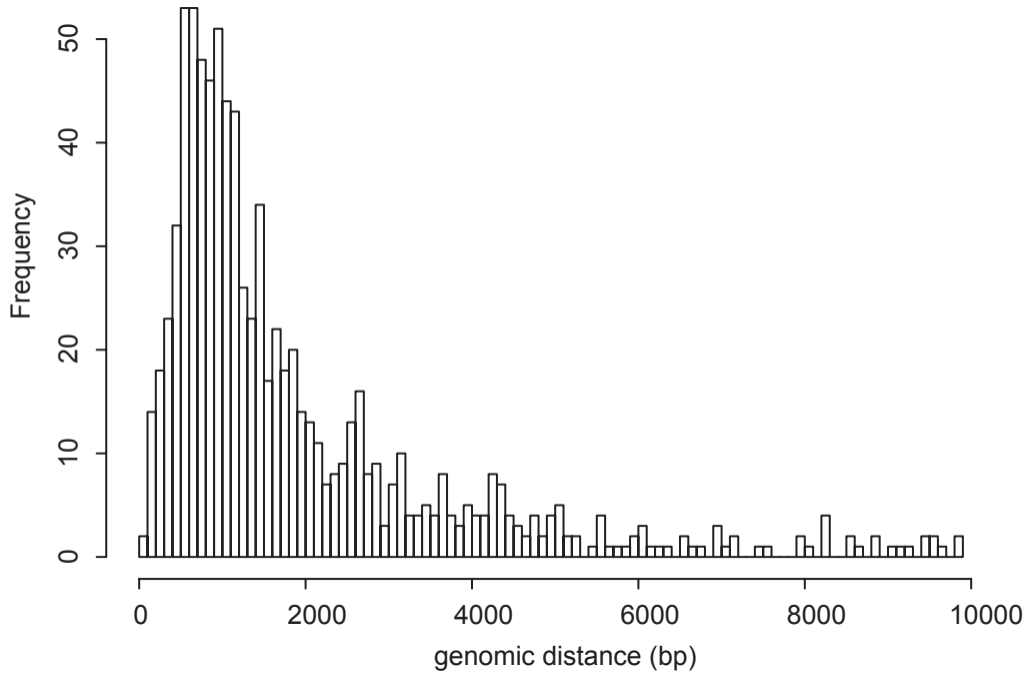

Supplement: Additional file 3 — Figure S2: Histogram of the distances between the primary and extended probe sets. The majority of predicted 3' UTR extensions are less than 2 kb. The graph is truncated at 10 kb but 5% of extensions are in the range of 10-500 kb. [file 1471-2164-11-205-S3.PDF]

chr7:

114734000

114734500

114735000

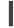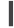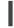

Olfml1

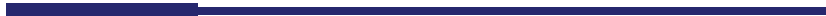

L1

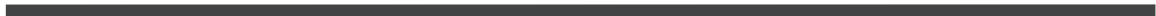

L2

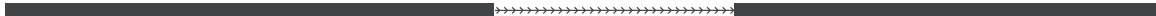

L3

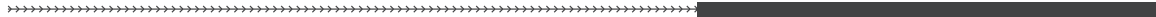

S1

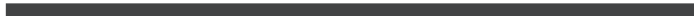

S2

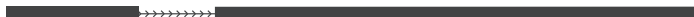

S3

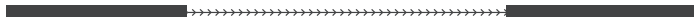

Supplement: Additional file 5 — Figure S3: Sequencing analysis of Olfml1 PCR products. Both S (short, known 3'UTR) and L (long, extended 3'UTR) products revealed 3 different alternative splicing forms, indicated as S1-S3 and L1-L3. S1 and L1 are most abundant as can be seen on Figure 3B. Note that all sequencing products start more upstream in the last but one exon of Olfml1; this region is not depicted and only the alignment with the 3'UTR is shown. [file 1471-2164-11-205-S5.PDF]

A

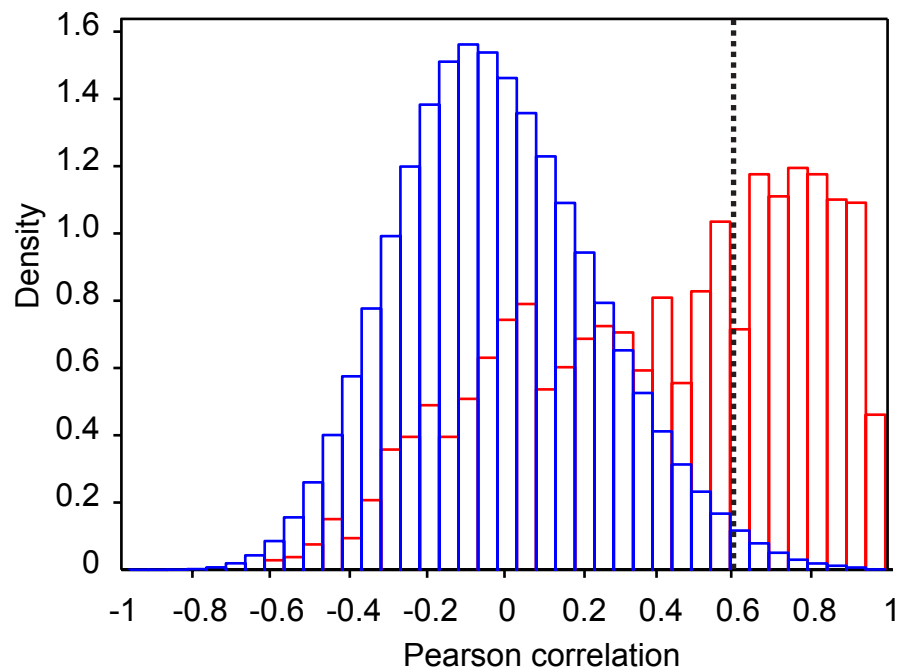

B

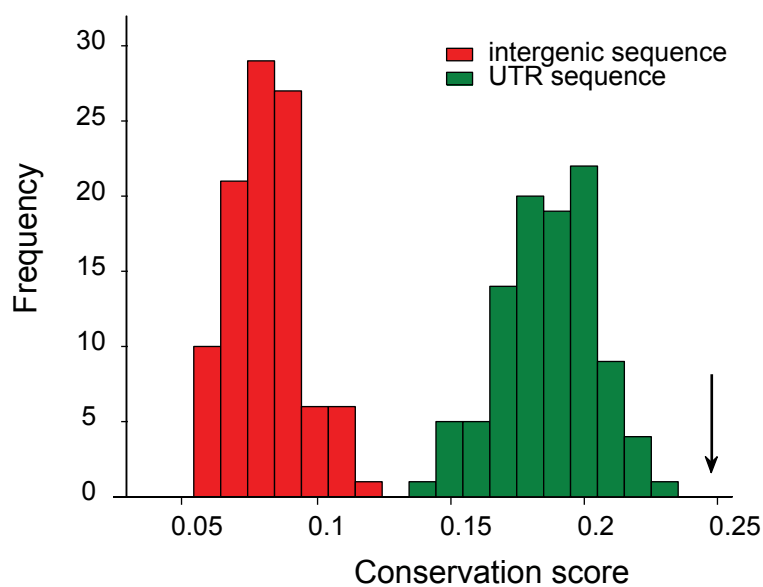

C

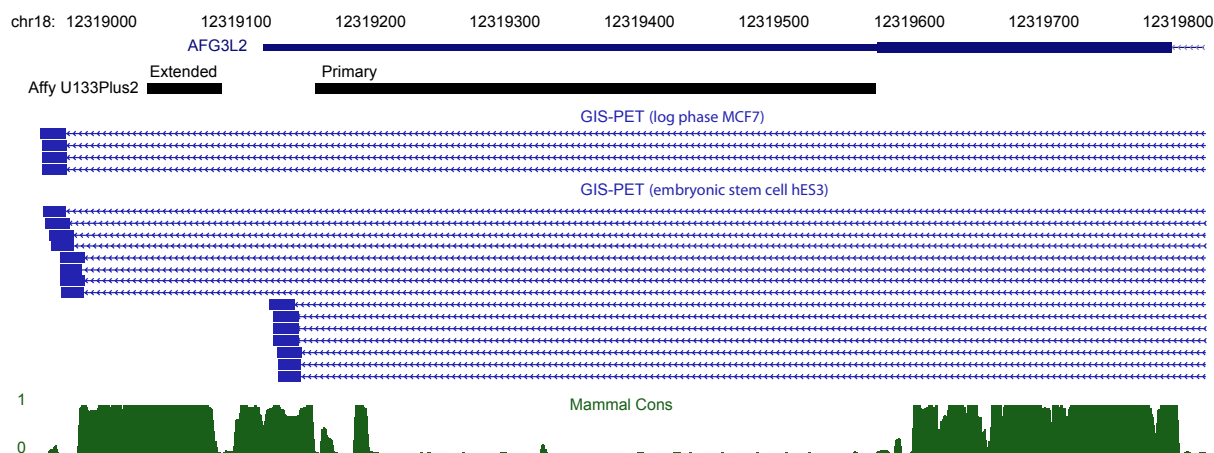

Supplement: Additional file 6 — Figure S4: Detection of extended 3' UTRs on the Affymetrix human U133 Plus 2.0 platform. A. Histogram with Pearson's correlations for human expression data. The red histogram depicts correlations between the 2126 probe set pairs before the final filtering step. The blue histogram depicts correlations between random probe sets. Similar to the mouse data, a Pearson correlation of 0.6 was chosen as a cut-off value. B. Human conservation score graphs, calculated with PhastCons based on the multiple alignment of 17 vertebrate species. Distributions in red and green represent intergenic and 3' UTR conservation respectively. Black arrow indicates the conservation score of the extended regions. C. GIS-PET track in the UCSC genome browser. GIS-PET tags are displayed in blue. Target regions for the Affymetrix U133 Plus 2.0 are indicated in black, PhastCons conservation score indicated in green. [file 1471-2164-11-205-S6.PDF]
